# Effects of Andresen Activator on Pharyngeal Airway Volume in Growing Patients With Obstructive Sleep Apnea Syndrome: CBCT Evaluation

**DOI:** 10.1155/ijod/9484241

**Published:** 2026-03-03

**Authors:** Lucia Giannini, Marco Farronato, Umberto Garagiola, Gianna Dipalma, Angelo Michele Inchingolo, Francesco Inchingolo, Cinzia Maspero

**Affiliations:** ^1^ Fondazione IRCCS Cà Granda Ospedale Maggiore Policlinico, Milan, 20122, Italy, policlinico.mi.it; ^2^ Dipartimento di Scienze Biomediche, Chirurgiche e Odontoiatriche, Università degli Studi di Milano, Milan, 20122, Italy, unimi.it; ^3^ Department of Interdisciplinary Medicine, School of Medicine, University of Bari “Aldo Moro”, Bari, 70124, Italy, uniba.it; ^4^ Department of Life Science, Health and Health Professional, Link Campus University, Roma, 00165, Italy

**Keywords:** Andresen activator, Class II, cone beam computed tomography, functional appliance, oropharyngeal airway, OSAS

## Abstract

**Aim:**

To assess the effects of Andresen functional appliance therapy on the pharyngeal airway passage (PAP) in growing Class II patients presenting with obstructive sleep apnea syndrome (OSAS). This update study incorporates new data from an extended sample and a revised follow‐up period to evaluate changes in airway dimensions using cone beam computed tomography (CBCT).

**Methods:**

Fifty growing patients with Class II malocclusion, mandibular hypoplasia, and OSAS, aged 9–14 (±1.63) years, were recruited for the study group. An additional 15 untreated patients (seven females and eight males) matched for age, sex, and craniofacial morphology served as the control group. Each patient had a CBCT performed before treatment (T0) and posttreatment after follow‐up (16–24 months‐T1). Pharyngeal airway dimensions were measured via medical 3D analysis software. The following measurements were recorded: depth of the oropharynx (DOP), depth of the hypopharynx (DHP), MP‐H (linear distance from mandibular plane to hyoid), posterior nasal spine (PNS)‐U (soft palate length), posterior airway space (PAS), SNA, SNB, and ANB. Statistical analysis (*t*‐tests for paired samples) was performed to compare T0–T1 changes within and between groups, with significance set at *p*  < 0.05.

**Results:**

This study showed the effects of the Andresen appliance on Class II correction, with an increase in SNB and a reduction in ANB. PAS increased by 2.94 mm (*p*  < 0.001). DOP and DHP showed improvements after expansion (*p*  < 0.001), and MP‐H decreased (3.62 mm; *p*  < 0.001), suggesting upward movement of the hyoid bone.

**Conclusions:**

In growing Class II patients, correction of mandibular retrusion using the Andresen appliance contributes to a sagittal increase in the oropharyngeal airway. These dimensional changes in the PAP also provided clinical improvements in sleep‐disordered breathing, supported by improved nocturnal polysomnographic (PSG) parameters. Such results might reduce anatomical and functional risk factors for pediatric OSAS. Functional orthopedic treatment performed during childhood has a role in airway patency promotion and long‐term OSAS risk decrease. A multidisciplinary approach is advised for optimal care.

## 1. Introduction

Obstructive sleep apnea syndrome (OSAS) is characterized by episodes of partial or complete obstruction of the upper airway, disrupting normal ventilation during sleep [1] and leading to specific clinical manifestations [2, 3].

The American Academy of Sleep Medicine defines OSAS based on the presence of at least five apnea–hypopnea events per hour of sleep in association with clinical symptoms, the most relevant being loud snoring and marked daytime sleepiness.

The therapeutic approaches for OSAS extend from conservative measures, including sleep hygiene (such as avoidance of alcohol and other substances, weight reduction, and appropriate sleeping posture) [4], to surgical interventions like glossectomy, uvulopalatopharyngoplasty, and maxillomandibular advancement [4–6]. Regardless of the chosen modality, treatment should aim to ensure patients maintain a good quality of life without adverse effects or significant risks [4].

In growing patients, mandibular retrusion and Class II malocclusion often correlate with reduced pharyngeal airway dimensions, contributing to signs and symptoms such as snoring, mouth breathing, and possible obstructive events [6]. Anatomical factors such as decreased oropharyngeal space, a retruded mandible, and low tongue posture can play a pivotal role in sleep‐disordered breathing. Several therapeutic modalities aim to improve airway patency in pediatric OSAS, including positional therapy, continuous positive airway pressure (CPAP), adenotonsillectomy, and functional appliances. In particular, functional appliances can reposition the mandible forward, thereby increasing the retrolingual space and potentially improving breathing [7–9]. Numerous studies have shown that functional appliances (e.g., Twin Block, Bionator, and Andresen activator) can enhance oropharyngeal dimensions, reducing apnea–hypopnea episodes and improving oxygen saturation [10, 11].

Among removable devices, the Andresen activator is widely used for skeletal Class II correction. It postures the mandible forward, guiding favorable growth at an optimal age (typically preadolescence to early adolescence). While widely employed for dentoskeletal correction, its effect on the upper airway and OSAS has drawn increased attention over the last decade [12, 13].

Measuring airway volume and dimensions precisely can be challenging. Traditional two‐dimensional (2D) radiographs such as lateral cephalograms present superimposed structures and can lead to errors. Cone Beam Computed Tomography (CBCT) addresses these limitations by providing a better display of the structures and the possibility to identify anatomical reference landmarks more precisely, both with point‐based measurements and also with more advanced volume and depth measurements. To ensure comparability with past literature on a larger sample and to provide an update, it was beyond the scopes of this study to evaluate volumes, so the study used traditional linear tracings traced on three‐dimensional images.

Despite growing evidence suggesting that functional appliances may improve upper airway dimensions in growing Class II patients, the available literature is still limited by studies with small sample sizes, heterogeneous study designs, and a lack of studies combining three‐dimensional airway assessment with objective polysomnographic (PSG) outcomes, especially involving patients with OSAS. Moreover, data specifically addressing the effects of the Andresen activator on both craniofacial morphology and pharyngeal airway dimensions in growing patients are very limited.

The aim of the present study was to evaluate the effects of Andresen functional appliance therapy on pharyngeal airway dimensions and skeletal relationships in growing Class II patients with OSAS. The null hypothesis was that Andresen appliance therapy does not produce significant changes in upper airway dimensions or PSG parameters compared with untreated controls.

## 2. Materials and Methods

### 2.1. Study Design and Ethical Approval

This retrospective longitudinal study was conducted in accordance with the Declaration of Helsinki and approved by the Ethics Committee of Azienda Ospedaliero‐Universitaria “Consorzio Policlinico” Bari n. 7593 of 25/01/2023. Written informed consent was obtained from participants’ parents or guardians. This study was reported in accordance with the STROBE guidelines for observational studies.

### 2.2. Sample Selection

A total of 50 patients with skeletal Class II malocclusion (ANB >4°; SNA ~82°; SNB <80°) and OSAS, aged 9–14 years, participated in this investigation (25 males, 25 females; mean age ~10.6 ± 1.6 years at T0). The control group consisted of 15 patients (seven females, eight males mean age ~11.05 ± 1.09 years at T0) matched for age, sex, and skeletal pattern but who did not undergo Andresen appliance therapy.

All patients were retrospectively selected from the clinical records of subjects treated at the Orthodontic Department, Fondazione IRCCS Cà Granda Ospedale Maggiore Policlinico, University of Milan, Italy and from Azienda Ospedaliero‐Universitaria “Consorzio Policlinico” Bari, Italy.

The control subjects did not undergo any orthodontic or orthopedic treatment during the observation period; their cone beam computed tomography (CBCT) scans at T0 and T1 were available from routine clinical monitoring and were included retrospectively.

#### 2.2.1. Inclusion criteria

• Class II skeletal relationship with normal to slightly protrusive maxilla and retrusive mandible.
• Age 9–14 years, at or before peak pubertal growth spurt.
• Documented OSAS, defined by nighttime polygraphic or PSG analysis revealing apnea–hypopnea index (AHI) ≥5 events/h or clinically relevant daytime sleepiness/snoring.
• No prior orthodontic or orthopedic treatment.

#### 2.2.2. Exclusion criteria

• Congenital syndromes or significant craniofacial anomalies.
• Previous maxillofacial surgery.
• Systemic conditions interfering with bone growth or muscle function.
• Noncompliance with scheduled appointments or poor appliance adherence.

### 2.3. CBCT Acquisition

All participants underwent CBCT at T0 (baseline) and T1 (after ~16 months). Scans were taken in a natural head position with the following parameters:

• 4 mA, 120 kV, 20 s scan time
• 0.3 mm voxel size, 16 cm × 22 cm field of view

Scans were exported in DICOM format and imported into Mimics 22 (Materialise, Leuven, Belgium). To reduce potential measurement bias, all CBCT analyses were performed independently by two calibrated examiners, and discrepancies were resolved by consensus with a senior examiner.

For both the treatment and control groups, CBCT scans were retrospectively retrieved from clinical records. The CBCT scans were originally prescribed for diagnostic purposes as part of routine orthodontic assessment and follow‐up and not for research aims. No additional CBCT scans were performed specifically for this study.

### 2.4. Cephalometric and Airway Analysis

Airway evaluation followed the protocol of Jena et al. [5] Key landmarks included:

• Posterior nasal spine (PNS)
• U (tip of the soft palate)
• V (vallecula)
• Ba (basion)
• H (hyoid)
• UPW = intersection of line Ptm–Ba with the posterior pharyngeal wall
• MPW = intersection of a perpendicular from U on Ptm–Ba with the posterior pharyngeal wall
• LPW = intersection of a perpendicular from V on Ptm–Ba with posterior pharyngeal wall

**Figure 1:**
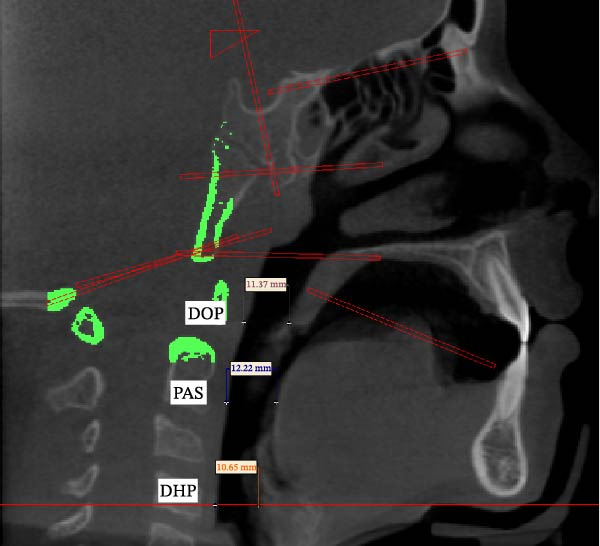
Illustrates the cephalometric landmarks and linear measurements used for dentoskeletal and airway analysis.

Standard dentoskeletal angles (SNA, SNB, and ANB) were recorded. Linear measurements for airway dimensions included:

• Oropharyngeal depth (DOP, distance from U to MPW) = DOP
• Hypopharyngeal Depth (DHP, distance from V to LPW) = DHP
• PNS–U = length of the soft palate
• PAS = minimal distance from the base of the tongue to the posterior pharyngeal wall on the B–Go extension
• MP–H = perpendicular distance from the mandibular plane (Go–Gn) to the hyoid (Figure 1).

### 2.5. Andresen Appliance Therapy

Patients in the treatment group were prescribed an Andresen activator fabricated by the same dental technician, with an incisal edge‐to‐edge wax bite registration. The vertical opening was set at 2–3 mm between maxillary and mandibular incisors, and the mandible was advanced to an incisor end‐to‐end relationship. Each patient was instructed to wear the appliance for at least 16 h daily, including nighttime. Appliance adjustments included selective grinding of the acrylic to allow vertical eruption while maintaining forward mandibular posture.

### 2.6. Updated PSG Evaluation

As part of this updated analysis, each participant had overnight polygraphic or PSG testing at T0 and T1. Apnea and hypopnea events were scored according to standard criteria from the American Academy of Sleep Medicine. The AHI was defined as the total number of apnea/hypopnea events per hour of sleep. An AHI <5 is generally considered a threshold for mild pediatric OSAS, though some clinicians may use a stricter cutoff.

### 2.7. Study Sample Size

The study sample consisted of all eligible patients who met the inclusion criteria during the study period.

### 2.8. Statistical Analysis

All measurements were retaken after a 1‐week interval to assess intraoperator reliability (Dahlberg’s formula). Statistical analyses were performed using a standard software package (SPSS v 25). Paired *t*‐tests compared T0 and T1 within each group; unpaired *t*‐tests compared differences between groups. A *p*‐value < 0.05 was considered significant, and *p*  < 0.001 was considered highly significant.

## 3. Results

### 3.1. Baseline Characteristics

At T0, the treatment group (*n* = 50) showed a mean SNB of 74.9° (SD ± 2.13), a mean ANB of 6.33° (±1.99), and a mean PAS of 3.74 mm (±0.87). Control subjects (*n* = 15) had similar initial measures: a mean SNB of 75.23° (±1.74), a mean ANB of 6.2° (±1.82), and a mean PAS of 3.58 mm (±0.51). Baseline polysomnography indicated a mean AHI of 12.4 events/h (±5.1) in the treatment group and 11.9 events/h (±4.9) in controls Table 1.

**Table 1:** Comparison of cephalometric and airway parameters (T0–T1) in patients and controls.

Patients	Age	SNA		SNB		ANB		MP‐H		PNS‐P (0)		PAS		DOP		DHP	
		T0	T1	T0	T1	T0	T1	T0	T1	T0	T1	T0	T1	T0	T1	T0	T1
SD	1.63	1.52	2.03	2.12	1.71	1.98	1.52	1.98	3.68	1.84	1.82	0.87	2.24	1.13	1.59	2.72	1.93
Average	10.69	81.13	81.50	74.90	77.26	6.33	4.47	28.24	24.62	43.22	42.70	3.76	6.68	4.65	6.29	9.90	12.02
Difference	—	0.370	—	2.360	—	−1.860	—	−3.620	—	−0.520	—	2.944	—	1.640	—	2.120	—
*p*‐Value	—	0.018	—	0.000	—	0.000	—	0.000	—	0.000	—	0.000	—	0.000	—	0.000	—

Controls	Age	SNA		SNB		ANB		MP‐H		PNS‐P (0)		PAS		DOP		DHP	
		T0	T1	T0	T1	T0	T1	T0	T1	T0	T1	T0	T1	T0	T1	T0	T1
SD	1.09	0.75	0.78	1.74	1.25	1.82	1.14	0.94	0.96	0.96	0.96	0.51	0.52	0.70	0.75	0.69	0.66
Average	11.05	81.43	81.55	75.23	75.68	6.20	5.87	28.20	28.27	43.27	43.27	3.58	3.60	5.21	5.31	11.14	11.23
Difference	—	0.11	—	0.45	—	−0.33	—	0.07	—	0.00	—	0.02	—	0.09	—	0.09	—
*p*‐Value	—	0.03	—	0.23	—	0.36	—	0.33	—	0.34	—	0.41	—	0.34	—	0.22	—
*p*‐Value pat vs. ctr	0.301	0.895	0.543	0.000	0.814	0.001	0.915	0.000	0.897	0.120	0.435	0.000	0.028	0.003	0.005	0.018	—

### 3.2. Skeletal Changes

After ~16 months:

• SNB in the treatment group increased significantly (from 74.9° ± 2.13 to 77.26° ± 1.72, *p*  < 0.001).
• ANB decreased (from 6.33° ± 1.99 to 4.47° ± 1.52, *p*  < 0.001), indicating successful Class II correction.
• SNA in the treatment group remained stable (81.13°–81.50°).
• Control subjects showed normal growth changes but without a large mandibular advancement (SNB from 75.23° to 75.68°; ANB from 6.2° to 5.87°).

### 3.3. Airway Dimensional Changes

• DOP: treatment group increased by + 1.64 mm (*p*  < 0.001); controls showed a minimal + 0.09 mm change (*p*  > 0.05).
• DHP: treatment group increased by + 2.12 mm (*p*  < 0.01); controls + 0.09 mm (*p* = 0.22).
• Posterior airway space (PAS): treatment group had an increase from 3.74 mm (±0.87) to 6.68 mm (2.94), *p*  < 0.001; the controls from 3.58 mm (±0.51) to 3.60 mm (±0.52), *p* = 0.41.
• MP–H (hyoid position): decreased by 3.62 mm in the treatment group (*p*  < 0.001), reflecting upward movement of the hyoid, whereas controls showed minor changes.
• PNS–U (soft palate length): slight decrease in the treatment group (from 43.22 mm ± 1.84 to 42.70 mm ± 1.82, *p* < 0.001); no significant change in controls.

### 3.4. PSG Outcomes

At T1, the treatment group’s mean AHI fell from 12.4 (±5.1) to 4.6 (±3.2), *p*  < 0.001. Controls changed from 11.9 (±4.9) to 10.2 (±5.0), *p* = 0.21. These findings suggest a clinically meaningful improvement in nocturnal breathing for patients using the Andresen appliance.

## 4. Discussion

Subjects with mandibular retrusion often display a small PAS and characteristic anatomical aspects of the soft palate [11, 12]. Orthopedic functional appliances for mandibular advancement have been shown to have a positive effect on the upper airway dimensions, as demonstrated in earlier studies and CBCT‐based investigations [13–17].

Of the imaging techniques available, CBCT can be considered a precise, reproducible, and reliable method of evaluating PAS dimensions [18].

In agreement with the present findings, CBCT has been described as a highly accurate and valuable diagnostic tool to assess airway morphology, and its reproducibility makes it particularly suitable for monitoring treatment‐related changes over time.

Results indicate that the Andresen activator favorably modifies both dentoskeletal relationships and the pharyngeal airway in growing Class II patients. Mandibular advancement appears to expand oropharyngeal and hypopharyngeal spaces, evidenced by significant increases in DOP, DHP, and PAS. The superior repositioning of the hyoid bone (MP–H) likely reflects forward tongue posture and reduced soft palate elongation, enhancing airway patency during sleep. The reciprocal distalizing effect on the maxilla and mandibular advancement, which promotes mandibular growth and causes an anterior repositioning of the tongue, can be used to explain the mechanism of action. This, in turn, results in an increase in airway caliber [19].

Recent biomechanical evidence further supports these clinical findings: using fluid–structure interaction simulation in a child with mandibular retrognathia, Wang et al. [20] demonstrated that Twin Block orthopedic treatment significantly reduced negative oropharyngeal pressure and airway wall deformation, suggesting an improved mechanical stability of the upper airway after mandibular advancement.

The concurrent improvement in AHI underscores that functional appliances can be an effective adjunct for mild to moderate pediatric OSAS. This aligns with other studies reporting that Class II correction can reduce snoring and apneic events by enlarging the retroglossal airway. Orthodontic collaboration remains crucial for the comprehensive treatment of pediatric patients with complex airway issues.

Recent randomized studies confirmed that functional appliances such as Twin Block and Myobrace can significantly improve sagittal pharyngeal airway dimensions in growing skeletal Class II patients through promoting mandibular advancement [21, 22].

In the untreated Class II control group, of which the PAS dimensions were analyzed in this study, there were only slight increases in PAS diameter. The rearward position of the tongue that is often found in mandibular retrusion patients has then caused the soft palate to be pushed backward, resulting in airway constriction [5]. This is in agreement with the results of Hänggi et al. [11], who did not observe any changes between adolescence in PAS, without treatment.

Results of the present study indicate assessments of changes in PAS dimensions from using the Andresen appliance. Analogous results have been found with other functional appliances [11, 12, 14–16]. Different previous studies reported improvement of PAS dimensions following functional appliance therapy in children and oral appliance therapy in adults [12–27].

These findings reinforce the hypothesis that early orthopedic correction of mandibular retrusion can counteract adaptive changes of the upper airway, thereby reducing risk factors predisposing to OSAS in adulthood.

Early treatment of growing patients with Class II malocclusion could prevent secondary modifications of the upper airway compensating for excessive horizontal growth and then resistance increasing, and decrease the risk of developing OSAS.

In addition, there is evidence in the literature for a long‐term stability of such airway changes after functional therapy [28, 29].

The treated group also showed an effective enrichment of sagittal jaw relation. Its effect is one of mandibular advancement with a concomitant restrictive or bland contrasting effect controlling maxillary growth, leading to forward mandibular development. Due to the advancement of the mandible, the tongue is moved forward, leading to the expansion of the sagittal dimension of the PAS. Comparable results have been observed in previous studies on activator treatment [30–34]. Cozza et al. [35] demonstrated that oral appliances may increase by 13% the collapsible area of the posterior pharyngeal wall in OSAS patients [35, 36].

In our study, mandibular advancement provided a significant improvement in oropharynx and hypopharynx depth and favorable inclination of the soft palate. Another study supported our hypothesis, Schutz et al. [37] showed that anterior positioning with the advancing of the mandible and hyoid bone after the correction of class II had produced forward traction of the tongue, increasing the posterior air passage by 3.2 mm and obstructing the airway in association with air resistivity.

The findings lend credence to the idea that functional appliances exert both orthopedic and functional effects, including a clinically significant improvement in airway patency and an increase in overall airway permeability.

## 5. Limitations

This study has some limitations that should be acknowledged.

First, its retrospective observational design may be associated with some selection bias and does not allow causal relationships.

Second, although the overall sample size was relatively large for a CBCT‐based study, the control group was smaller, and this may limit the statistical power of between‐group comparisons (it is not easy to identify a large control group with appropriate CBCT scans).

Third, airway assessment was based on linear CBCT measurements rather than full volumetric analysis, which might not completely reflect three‐dimensional airway changes. CBCT images were acquired in an awake, upright position, which may not fully represent airway behavior during sleep. Although polysomnography demonstrated significant clinical improvement, long‐term follow‐up after completion of growth was not available; therefore, the stability of airway changes over time remains to be clarified.

Future prospective randomized studies with larger control samples, volumetric airway analysis, and longer follow‐up are warranted to confirm and extend these findings.

## 6. Conclusions

We can conclude as follows:

– Andresen functional appliance therapy in growing skeletal Class II patients with OSAS leads to a significant improvement in sagittal maxillo–mandibular relationships, with an increase in SNB angle, and a reduction in ANB angle.
– Orthopedic treatment is usually associated with a significant improvement in airway dimensions, as demonstrated by increases in PAS, DOP, and DHP, together with a favorable superior repositioning of the hyoid bone.
– These anatomical changes lead to a clinically meaningful reduction in the apnea index, indicating an improvement in nocturnal breathing.
– Given the multifactorial nature of pediatric OSAS, orthodontic management should be integrated within a multidisciplinary team, including PSG records and ENT collaboration and sleep medicine specialists.

These findings support the role of early functional orthopedic treatment in promoting upper airway changes, and potentially reducing anatomical risk factors for pediatric obstructive sleep apnea.

## Author Contributions

Conceptualization: Cinzia Maspero, Marco Farronato, and Lucia Giannini. Methodology: Lucia Giannini, Gianna Dipalma, and Angelo Michele Inchingolo. Formal analysis, data curation, visualization, writing – original draft: Marco Farronato and Lucia Giannini. Investigation: Cinzia Maspero and Marco Farronato. Resources: Gianna Dipalma, Marco Farronato, Lucia Giannini, and Umberto Garagiola. Software: Umberto Garagiola. Writing – review and editing: Marco Farronato, Lucia Giannini, and Cinzia Maspero. Supervision, project administration: Cinzia Maspero and Francesco Inchingolo. Funding acquisition: Cinzia Maspero, Francesco Inchingolo, and Gianna Dipalma. Visualization, writing – original draft: Lucia Giannini and Marco Farronato. All authors should have made substantial contributions to all of the following: (1) the conception and design of the study, or acquisition of data, or analysis and interpretation of data and (2) drafting the article or revising it critically for important intellectual content.

## Funding

The authors have nothing to report. Open access publishing facilitated by Universita degli Studi di Milano, as part of the Wiley ‐ CRUI‐CARE agreement.

## Disclosure

All authors have made substantial contribution to the final approval of the version to be submitted.

## Ethics Statement

This study was conducted in accordance with the Declaration of Helsinki and approved by the Ethics Committee of Azienda Ospedaliero‐Universitaria “Consorzio Policlinico” Bari n. 7593 (of 25/01/2023).

## Consent

Written informed consent was obtained from all patients for use of their clinical data for research and publication purposes.

## Conflicts of Interest

The authors declare no conflicts of interest.

## Data Availability

The data presented in this study are available upon reasonable request, after the signature of a formal data sharing agreement in anonymous form, from the corresponding author, because they are protected by privacy.
